# Cannabis Smoking-induced Diffuse Alveolar Hemorrhage

**DOI:** 10.7759/cureus.5089

**Published:** 2019-07-06

**Authors:** Ali Alqahtani, Zaid Ammari, Ahmad Ramahi, Tamer S Said Ahmed, Emile Klada

**Affiliations:** 1 Internal Medicine, University of Toledo Medical Center, Toledo, USA; 2 Pulmonary / Critical Care Medicine, University of Toledo Medical Center, Toledo, USA; 3 Pulmonary / Critical Care Medicine, Promedica Toledo Hospital, Toledo, USA

**Keywords:** diffuse alveolar hemorrhage, cannabis

## Abstract

Cannabis smoking is common among adolescents and young adults. Diffuse alveolar hemorrhage (DAH) is a rare and life-threatening complication of cannabis abuse. DAH is characterized by bleeding into alveoli secondary to the disruption of the alveolar-capillary basement membrane as a result of an injury at the level of alveolar microcirculation. The differential diagnosis of DAH includes systemic vasculitis, bland pulmonary hemorrhage, and alveolar damage. The impact of cannabis on the respiratory function includes mucus hypersecretion, inflammatory edema, and increased alveolar permeability. Moreover, in vitro coagulation studies on rats showed that two major cannabinoids, cannabinol and THC, have antithrombotic activity. We present two cases of cannabis use resulting in acute lung injury and diffuse alveolar hemorrhage.

## Introduction

Diffuse alveolar hemorrhage (DAH) is a severe disorder, which manifests with hemoptysis and diffuse alveolar infiltrates, as well as hypoxemia respiratory failure [1]. DAH originates from the pulmonary microcirculation, including the alveolar capillaries, arterioles, and venules, and is usually diffuse but may also be focal [2]. The etiologies of DAH are generally divided into autoimmune and non-autoimmune causes, which include malignancy, infections, coagulation disorders, cardiovascular diseases, and illicit drug use [3].

Illicit substance abuse can cause multiple pulmonary manifestations that relate to specific agents as well as their mode of delivery, chronicity of use, and the presence of additives [4]. Cannabis is the most commonly used inhaled drug in the United States after tobacco, with an estimated 22.2 million people ages 12 years and older reporting current use [5]. Smoking cannabis has been linked to many side effects, including pneumothorax, pneumomediastinum, and subcutaneous emphysema [6]. Most reports often involved cocaine usage as a major cause of DAH, with little reports linking it to cannabis usage. Here, we present a case and re-present another case (Poster presentation: Ammari Z, Rehman S, Hernandez DA: Cannabis use resulting in diffuse alveolar hemorrhage: a diagnosis of exclusion. Chest. Annual Meeting. October 25, 2016) of cannabis use resulting in acute lung injury and DAH.

## Case presentation

Case report one

**Figure 1:**
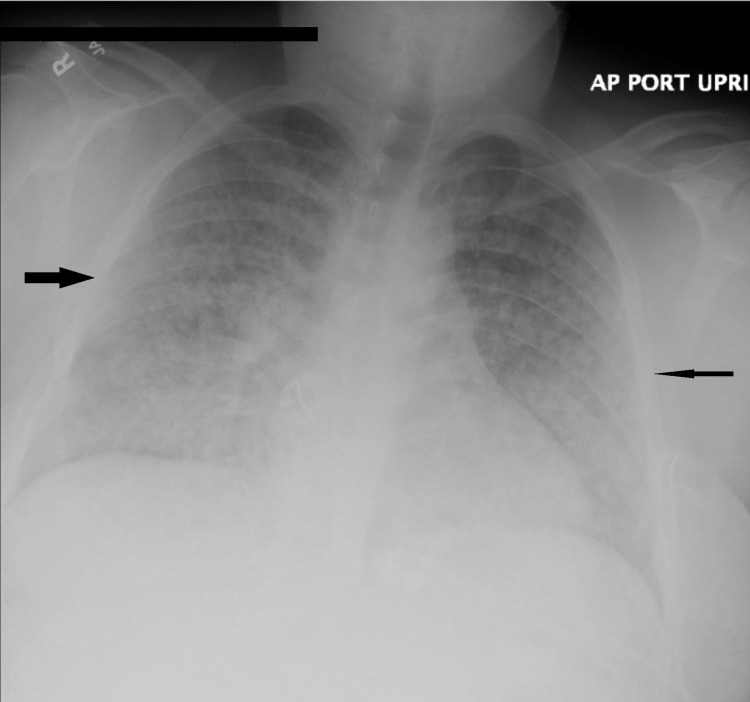
Chest X-ray shows diffuse pulmonary infiltrates (arrows)

A 19-year-old Hispanic male with a past medical history significant for seizure, bipolar disorder, and marijuana abuse was admitted to the hospital after a witnessed acute onset seizure. He presented to the emergency room (ER) and was found to be in acute distress and coughing up blood. His vital signs were: blood pressure 170/78 mmHg, pulse 140 bpm, respiratory rate 32/minute, with an oxygen saturation of 82% in ambient air, for which he was placed on non-rebreather oxygen. He was alert and oriented to time, person, and place. His respiratory exam revealed diminished air entry bilaterally, with diffuse rhonchi. The remainder of his exam was unremarkable. Arterial blood gas (ABG) showed pH 7.139, PCO_2_ 43.1, PO_2_ 86, and HCO_3_ 14.6. Chest X-ray (CXR) showed diffuse pulmonary infiltrates (Figure 1). The patient was intubated, sedated, and transferred to the ICU. He reported smoking two to five non-synthetic marijuana joints per day (though he denied the use of any inhalant aid, i.e. bongs) and had no significant occupational exposures or other illicit drug use.

**Figure 2:**
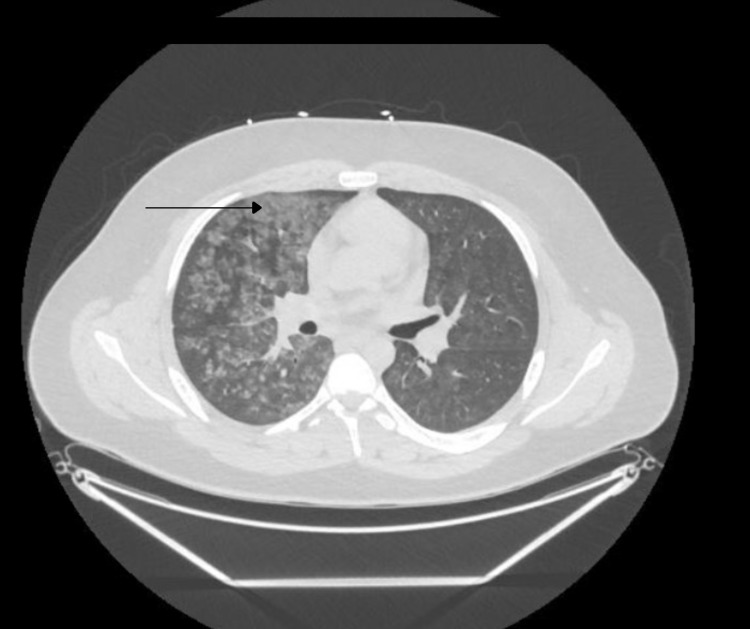
Chest CT scan with no contract showed diffuse ground-glass opacities (arrow) throughout the right lung and, to a lesser extent, in the left lung

His initial laboratory evaluation included hemoglobin of 16.4 g/dl, leukocytosis of 22.4 k/μL (80% neutrophils), and normal platelet count. Renal function, liver function, electrolytes, as well as coagulation studies, were all normal. The urine toxicology screen was positive for tetrahydrocannabinol (THC) and negative for other illicit drugs, and urinalysis showed no red blood cells (RBCs) or white blood cells (WBCs). Chest computed tomography (CT) without contrast verified the CXR findings and showed diffuse ground-glass opacities throughout the right lung and, to a lesser extent, in the left lung (Figure 2).

The patient was started on empiric antibiotics and steroids with ceftriaxone, azithromycin, and methylprednisolone. Bronchoalveolar lavage (BAL) was successfully performed and revealed increased hemorrhagic patterns on serial BAL. Cell counts were remarkable for an RBC of 12265 cells/μl with 380 white cells/μl (47% macrophages). Cytology showed abundant hemosiderin-laden alveolar macrophages. BAL bacterial, viral, fungal, acid-fast bacilli (AFB), and Pneumocystis jirovecii cultures were negative. The infectious workup, including blood and sputum cultures, legionella urinary antigen, respiratory viral panel, quantiferon tuberculosis (TB) test, and human immunodeficiency (HIV) antibodies, was negative. Echocardiogram was normal, with no structural and functional pathologies.

Extensive rheumatological and vasculitis serologic evaluation, including erythrocyte sedimentation rate (ESR), C-reactive protein (CRP), antinuclear Ab, antineutrophil cytoplasmic Ab, glomerular basement membrane Ab, cryoglobulins, antiphospholipid Ab, and rheumatoid factor, were all negative.

The patient's condition improved and after extubation on his third day of stay, he left the hospital against medical advice. The patient was admitted multiple times to the hospital afterward with a similar presentation and continues to smoke marijuana despite counseling.

Case report two

**Figure 3:**
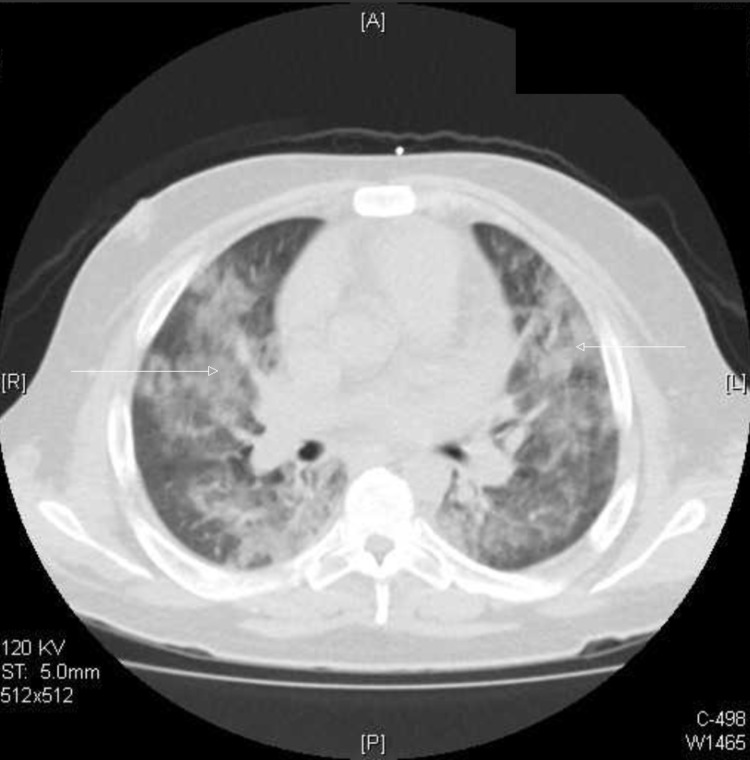
Chest CT scan shows diffuse bilateral patchy opacities (arrows) CT: computed tomography

**Figure 4:**
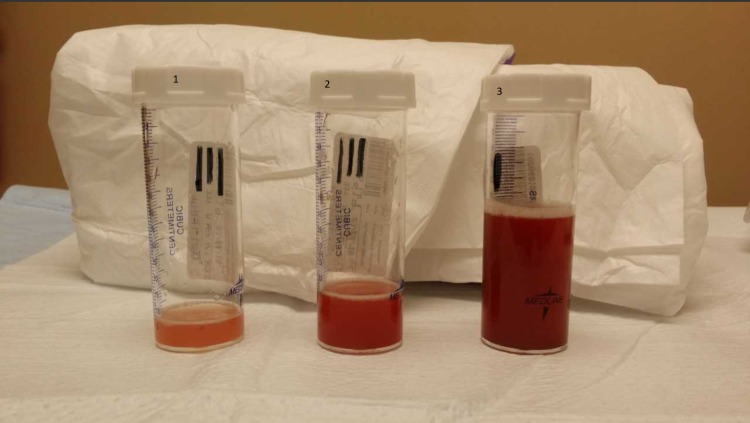
Bronchoscopy demonstrated increased hemorrhagic patterns on serial bronchoalveolar lavage

A 33-year-old Hispanic male presented to our institute with dyspnea. His past medical history was significant for diabetes mellitus type I and end-stage renal disease (ESRD) on hemodialysis. He reported recent use of cannabis. The patient was in mild respiratory distress, afebrile, with oxygen saturation of 84% on room air. The physical exam was positive for diffuse rales. Arterial blood gas revealed low pO_2_ and high A-a gradient. Laboratory workup revealed hemoglobin 8.7g/dl, WBC count 9800/mm^3^, platelets 387000/mm^3^, and international normalized ratio (INR) 1.1. Chest radiograph and CT scan showed diffuse bilateral patchy opacities (Figure 3). Bronchoscopy demonstrated increased hemorrhagic patterns on serial BAL (Figure 4). Cytology showed abundant hemosiderin-laden alveolar macrophages. BAL bacterial, viral, fungal, AFB, and Pneumocystis jirovecii cultures were negative. Rapid influenza test, anti-streptococcal antibody (Ab), and Legionella urinary antigen were negative.

Echocardiogram revealed normal ejection fraction with no mitral stenosis. Antinuclear Ab, antineutrophil cytoplasmic Ab, glomerular basement membrane Ab, cryoglobulins, antiphospholipid Ab, and rheumatoid factor were all negative. The serum toxicology screen performed on admission was positive for THC with a value of 163 ng/ml (cutoff: 5 ng/mL) and negative for cocaine. The patient left the hospital against medical advice and presented in five days with hemoptysis and worsening dyspnea. There was a drop in hemoglobin to 7.1 g/dl. He was transfused one unit of packed red blood cells and managed supportively with supplemental oxygen (Poster presentation: Ammari Z, Rehman S, Hernandez DA: Cannabis use resulting in diffuse alveolar hemorrhage: a diagnosis of exclusion. Chest. Annual Meeting. October 25, 2016).

## Discussion

The diffuse alveolar hemorrhage (DAH) disorder is a life-threatening condition caused by a variety of disorders associated with hemoptysis, anemia, diffuse lung infiltration, and acute respiratory failure and is linked with a high rate of mortality [2]. The clinical presentation is important in diagnosing DAH but bronchoscopy with BAL is the gold standard for DAH diagnosis.

DAH and pulmonary complications have been associated with illicit drug use, including cocaine, amphetamine, and cannabis smoking [7]. Cannabis smoking has been known to be associated with histological evidence of airway inflammation, edema, and increased alveolar permeability. The mechanism of cannabis-induced DAH is unclear but deep inhalation of marijuana has been associated with pneumothorax, pneumomediastinum, and subcutaneous emphysema. However, the same mechanism can increase the risks of DAH considering the increased negative alveolar pressure, which can worsen the damage to the alveolar-capillary membrane [8]. Moreover, in vitro coagulation studies showed the antithrombotic activity of cannabis by extract on thrombin activity and inhibition of thrombin-induced clot formation [9].

Our two cases were admitted with hemoptysis. They developed blood-streaked sputum from excessively smoking marijuana joints. Both showed no significant exposure to other illegal drugs except for non-synthetic marijuana. Moreover, there was consistency with DAH, when bronchoalveolar lavage (BAL) tests were performed. Two other French cases of DAH resulting from marijuana smoking using plastic bongs were reported. In these cases, incomplete combustion between plastic and marijuana released acid anhydrides, causing alveolar hemorrhage. These two patients had smoked marijuana for 15 years, and it was a certainty that excessive marijuana caused DAH, considering there was no other etiology [10]. In conclusion, smoking marijuana might cause alveolar hemorrhage, which could be life-threatening.

## Conclusions

Here, we presented two rare cases of cannabis use resulting in acute lung injury and DAH. Clinicians should have a high suspicion for substance abuse in young patients presenting with DAH. These two cases highlight the potentially fatal complication of DAH in subjects smoking cannabis. Further investigation about cannabis smoking and DAH are warranted.
